# Expression of αA-crystallin (CRYAA) *in vivo* and *in vitro* models of age-related cataract and the effect of its silencing on HLEB3 cells

**DOI:** 10.18632/aging.204754

**Published:** 2023-05-28

**Authors:** Xiaoling Ma, Yi Nan, Can Huang, Xiangyang Li, Yifan Yang, Wenjie Jiang, Mengyi Ye, Qian Liu, Yang Niu, Ling Yuan

**Affiliations:** 1 Ningxia Medical University Key Laboratory of Ningxia Minority Medicine Modernization Ministry of Education, Ningxia Medical University, Yinchuan 750004, Ningxia, China; 2 School of Traditional Chinese Medicine, Ningxia Medical University, Yinchuan 750004, Ningxia, China; 3 School of Clinical Medicine, Ningxia Medical University, Yinchuan 750004, Ningxia, China; 4 School of Pharmacy, Ningxia Medical University, Yinchuan 750004, Ningxia Hui Autonomous Region, China

**Keywords:** age-related cataract, CRYAA

## Abstract

Aim: To investigate the expression of αA-crystallin (CRYAA) in age-related cataract (ARC) models and its role in lens epithelial cells (LECs).

Methods: We used Flow cytometry to detect the apoptosis and cell cycle in HLEB3 cells and Real-time fluorescence quantitative polymerase chain reaction to detect the expression of CRYAA mRNA in HLEB3 and in rabbit lens. The expression of CRYAA in HLEB3 cells and rabbit lenses as well as the proteins related to apoptosis and autophagy in transfected cells were detected by western blotting. The lens structure in rabbits was investigated using hematoxylin-eosin staining. Protein thermostability assay was performed to detect the thermal stability of rabbit lens proteins. CCK- 8 assay was used to detect the viability of transfected cells, and the transfection was recorded by fluorescence photography.

Results: Hydrogen peroxide can promote apoptosis and arrest the cell cycle in HLEB3 cells, and naphthalene can cause cataract formation and damage the structure of the lens in rabbits. Both ARC models can reduce the expression of CRYAA. The expression of CRYAA silencing increased apoptosis and autophagy in HLEB3 cells.

## INTRODUCTION

Age-related cataract (ARC), a multifactorial disease, is the leading cause of visual impairment and blindness worldwide. The main pathogenic component of ARC is oxidative damage and aging [1].

Reactive oxygen species include non-free radicals like hydrogen peroxide (H_2_O_2_), which can best produce oxidative damage and lead to irreversible apoptosis in lens epithelial cells (LECs) [2], opacifying the lens and even causing blindness. Therefore, a well-known model for examining the connection between oxygen free radicals and cataract is the LECs’ H_2_O_2_ damage model *in vitro* [3]. Cataract induced by naphthalene is more common *in vivo* experiments. This cataract model displays good concordance with age-related cataract in terms of the oxidative damage mechanism and its early morphology. For example, naphthalene and its metabolites during the metabolic process can cause oxidative damage, which develop cataract, and its treatment caused a modest increase in the amount of light scattering in the mouse lens’ nuclear area, which is comparable to the pattern created in the human ARC lens [4], and its proteome level is also similar to ARC [5]. And the morphological and the pathophysiological changes of naphthalene-induced cataract to genesis are reported to be very similar to ARC [6–8]. Hence, naphthalene cataract can be utilized as an animal model to research the development of age-related cataract.

Protein precipitation and aggregation, caused by insufficient crystallin stability, can lead to hazy lenses and the formation of ARC [9]. The αA-crystallin (CRYAA) has a significant impact on preserving the lens' clarity. As a highly conserved cytoskeletal protein with chaperone-like activity (CLA), CRYAA can prevent the hyper-aggregation of other lens proteins, such as β/γ-crystallin, which forms a heterooligomer complex known as α-crystallin with CRYAA in 1:3 ratio [10–12]. Additionally, CRYAA can enhance the resistance of tissues and cells to oxidative stress [13]. Autophagy is a critical requirement for lens cell remodeling, organelle degradation, and transparency [14]. However, the continued exposure chronic stress with advancing age can reduce the fidelity of the autophagy-lysosomal system, which recognizes and removes damaged proteins [15].

In order to evaluate the expression of CRYAA in ARC, and the relationship between CRYAA silencing and apoptosis and autophagy, we decided to conduct this study using two comparative cataract modeling techniques: *in vitro* (hydrogen peroxide cataract) and *in vivo* (naphthalene cataract).

## RESULTS

### H_2_O_2_ promoted cell apoptosis and arrested cell cycle in HLEB3

**Figure 1:**
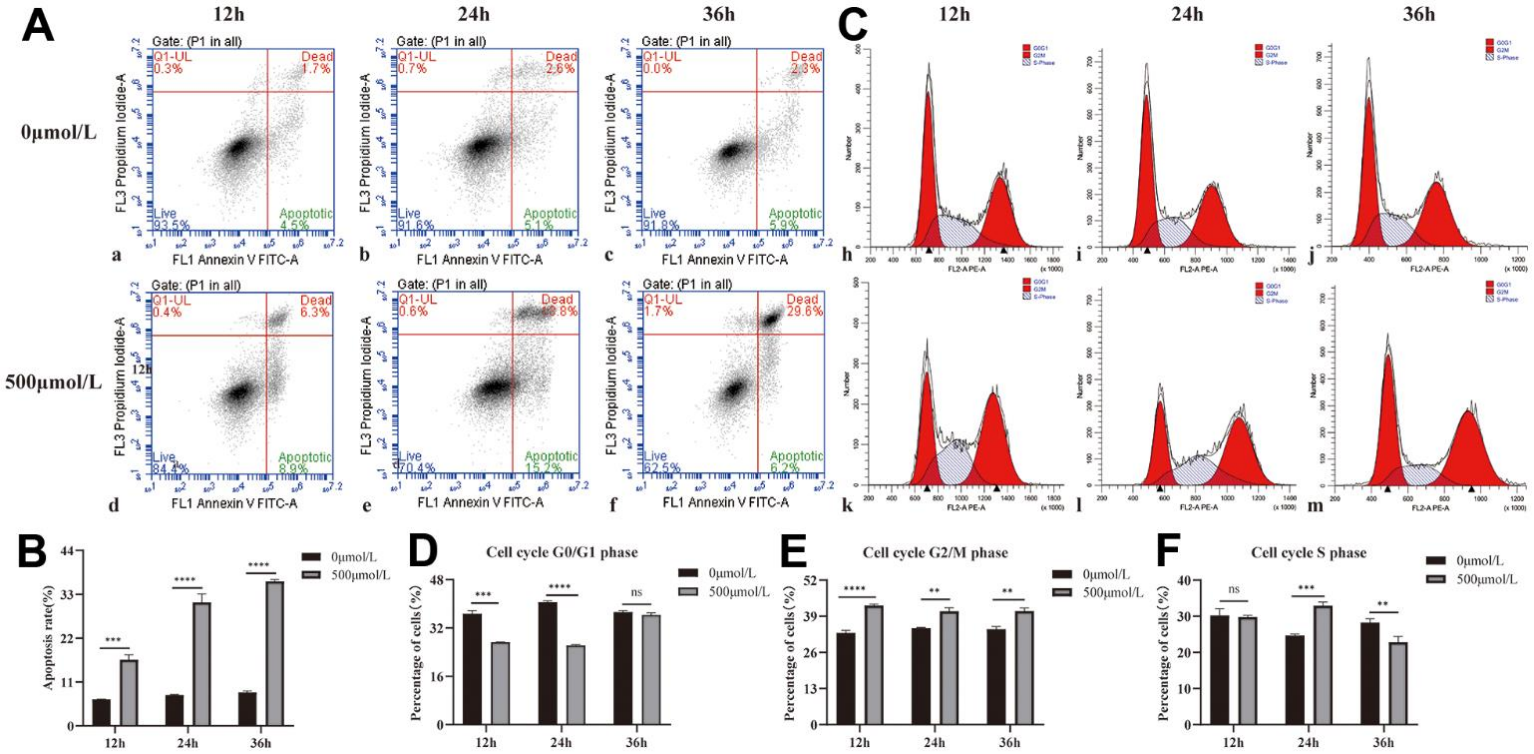
**Effect of H_2_O_2_ on apoptosis and cycle of HLEB3.** (**A**) a, b, and c are the 0μmol/L H_2_O_2_ group apoptotic diagrams at 12, 24, and 36 hours, and d, e, and f are the 500μmol/L H_2_O_2_ group apoptotic diagrams at 12, 24, and 36 h, respectively. (**B**) The statistical result of apoptosis in each group. (**C**) h, i and j are the 12, 24 and 36h cycle diagrams of the 0μmol/L H_2_O_2_ group, and k, l and m are the 12, 24 and 36 h cycle diagrams of the 500μmol/L H_2_O_2_ group, respectively. (**D**–**F**) The statistical results of each period. ns *p*>0.05, **p<0.01, ***p<0.001, ****p<0.0001.

Based on the apoptosis results of 0 and 500μmol/L H_2_O_2_ for 12, 24, and 36 hours (Table 1 and Figure 1A, 1B), the apoptosis rate of HLEB3 cells in the 500μmol/L H_2_O_2_ group significantly over time compared to the 0μmol/L H_2_O_2_ group. These findings suggest that hydrogen peroxide promotes HLEB3 cells to undergo apoptosis, with the rate of apoptosis increasing the most at 24 hours. The statistical analysis showed that the results were significant across all three time periods.

**Table 1:** The result of cell apoptosis.

**Time (h)**	**Group**	**Apoptosis rate (%)**	**Increase rate of apoptosis (%)**	***P* value**
12h	0μmol/L H_2_O_2_	6.5%±0.22%	10.10%±0.79%	0.0001
	500μmol/L H_2_O_2_	16.60%±0.99%		
24h	0μmol/L H_2_O_2_	7.73%±0.12%	14.37%±1.02%	<0.0001
	500μmol/L H_2_O_2_	30.97%±1.72%		
36h	0μmol/L H_2_O_2_	8.40%±0.28%	5.30%±1.42%	<0.0001
	500μmol/L H_2_O_2_	36.27%±0.34%		

The G2/M phase of the 500μmol/L H_2_O_2_ group was higher compared to the 0μmol/L H_2_O_2_ group at all three time points, indicating that hydrogen peroxide could arrest the cell cycle of HLEB3 cells in G2/M phase. The cell cycle results of 0 and 500μmol/L H_2_O_2_ at 12, 24 and 36 hours are presented in Table 2 and Figure 1C–1F. The S phase of the HLEB3 cycle grew dramatically and was arrested in the H_2_O_2_ group at 24 hours, showing that 500μmol/L H_2_O_2_ had the most pronounced effect on cell cycle arrest at that time.

**Table 2:** The result of cell cycle.

	**Cell cycle phase**	**C**	**M**	***P* value**
12h	G0/G1	36.74%±0.86%	27.31%±0.86%	0.0001
	G2/M	33.02%±0.74%	42.90%±0.38%	<0.0001
	S	30.24%±1.50%	29.79%±0.37%	0.1102
24h	G0/G1	40.54%±0.32%	26.20%±0.22%	<0.0001
	G2/M	34.71%±0.21%	40.86%±1.00%	0.001
	S	24.74%±0.24%	32.94%±0.80%	0.0002
36h	G0/G1	37.31%±0.42%	36.33%±0.53%	0.7012
	G2/M	34.43%±0.71%	40.88%±0.87%	0.0013
	S	28.26%±0.87%	22.8%±1.35%	0.0085

### H_2_O_2_ reduced the expression of CRYAA in HLEB3

**Figure 2:**
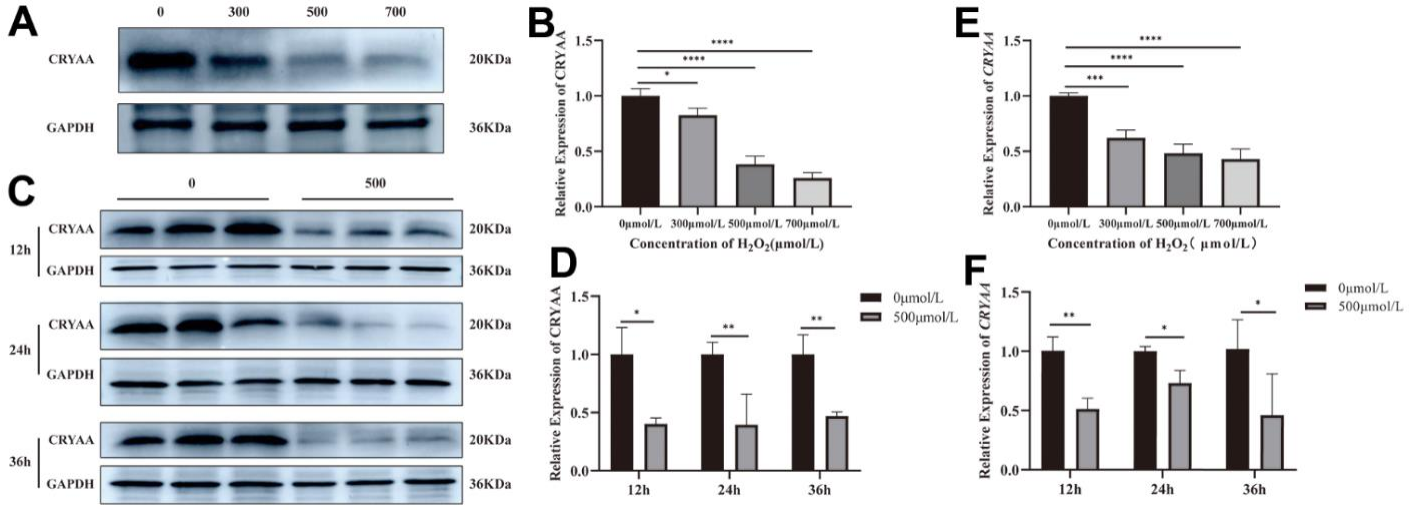
**CRYAA expression *in vivo* experiments.** (**A**, **B**) The expression of CRYAA protein in HLEB3 cells treated with 0, 300, 500, 700μmol/L H_2_O_2_ for 24 hours and (**C**, **D**) 0, 500μmol/L H_2_O_2_ for 12, 24, 36 hours. (**E**) The expression of *CRYAA* mRNA in HLEB3 cells treated with 0, 300, 500, 700μmol/L H_2_O_2_ for 24 hours; (**F**) *CRYAA* mRNA expression in HLEB3 cells treated with 0 and 500μmol/L H_2_O_2_ for 12, 24, and 36 hours. ns *p*>0.05, *p<0.05, **p<0.01, ***p<0.001.

Compared with the normal group, the results of *CRYAA* mRNA and protein expression in cells treated with 300, 500, and 700μmol/L H_2_O_2_ for 24 hours are respectively shown in Figure 2E (*p* value: 0.0233, <0.0001, <0.0001) and Figure 2A, 2B (*p* values: 0.328, 0.0005, 0.0001). Both mRNA and protein expression levels decreased. After intervention with 0 and 500μmol/L H_2_O_2_ for 12, 24, and 36 hours, the expression levels of *CRYAA* mRNA (Figure 2F (*p* values: 0.0005, <0.0001, <0.0001)) and CRYAA protein (Figure 2C, 2D (*p* values: 0.0046, 0.0182, 0.0452)) of 500μmol/L H_2_O_2_ group all decreased compared with the normal group. The results showed an overall decrease in the expression of the *CRYAA* mRNA and protein expression in HLEB3 cells after H_2_O_2_ treatment.

### Modeling and material extraction

**Figure 3:**
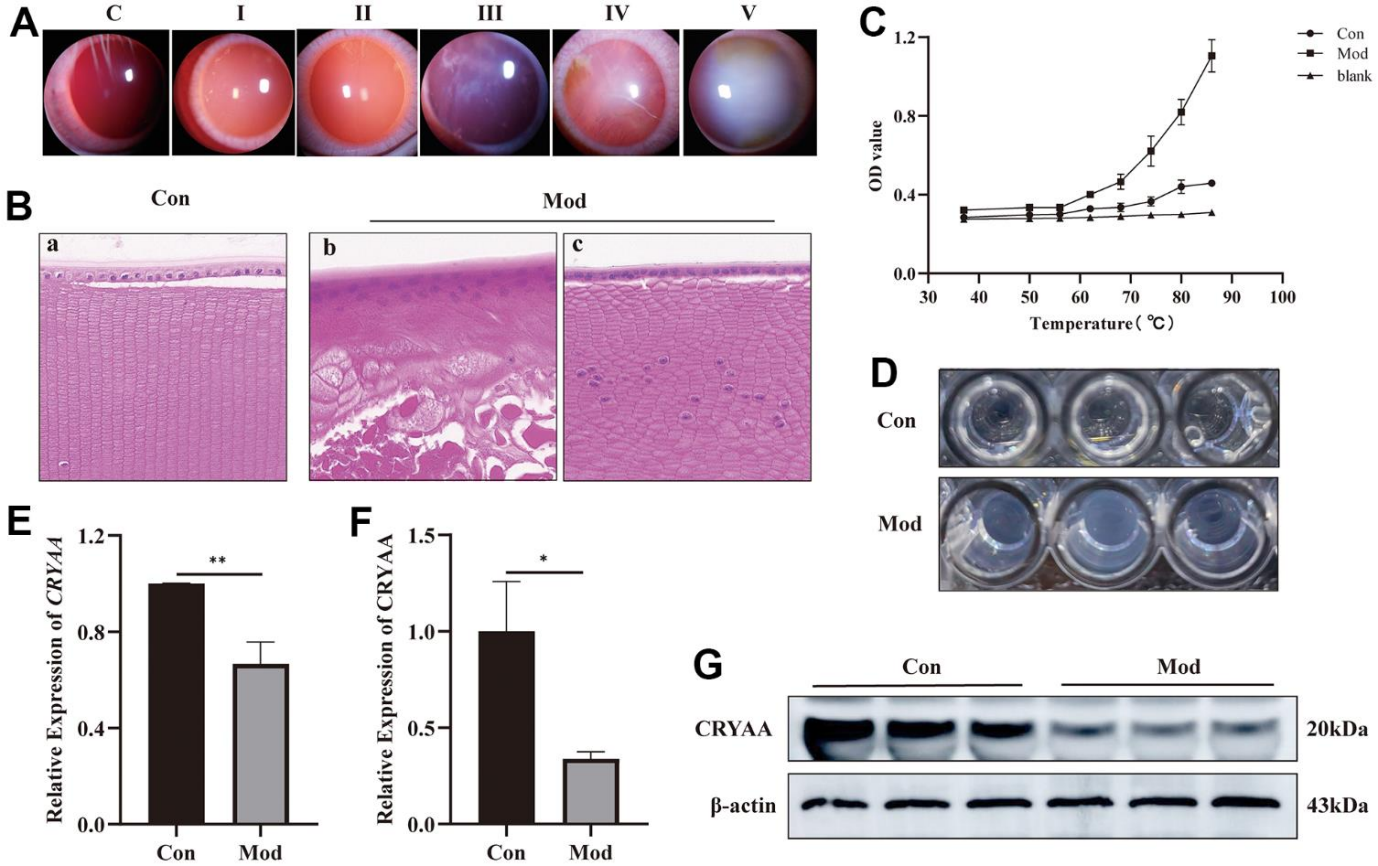
**Experimental results of influence of naphthalene on rabbit lens.** (**A**) Slit-lamp images of all grades of rabbit cataract; (**B**) The normal group (a) and model group (b, c) of rabbit lens HE staining (400x); (**C**) Statistical results of protein thermal stability experiment; (**D**) The picture of protein supernatant at 86° C; (**E**) Expression of *CRYAA* mRNA in rabbit lenses, **p<0.01; (**F**) Expression of CRYAA protein in rabbit lenses, *p<0.05; (**G**) The result of WB banding of CRYAA in rabbit lenses.

Following naphthalene treatment, slit-lamp examinations were performed to evaluate the condition of the rabbits’ lenses. Within three weeks, small vesicles began to emerge around the lenses, prompting the cessation of the naphthalene treatment and the resumption of a normal diet. Subsequent slit-lamp observations were conducted every 3-4 days to monitor the progression of cataract formation, with corresponding images and timestamps recorded according to the grading standard illustrated in Figure 3A. Additional information, including the development time and grading criteria, is presented in Table 3 [16].

**Table 3:** Evolution and classification of lenticular opacity and time of development.

**Cataract stage (stage)**	**Degree of lens opacity**	**Development time (weeks)**
I	Small vesicles, or groups of small vesicles, begin to develop on the periphery of the lens after mydriasis.	3
II	The peripheral vacuoles of the lens gradually develop a ring- or ring-like opacities in the peripheral or posterior cortex after mydriasis, which have no effect on the ability to see the fundus.	5
III	The fundus details are obscured by a grayish-white opacity that forms in the cortical region of the lens in the absence of mydriasis.	6
IV	The cortical region of the lens is clouded and fused; crescent-shaped projections or water fades are seen there, but no fundus features are visible.	6.5
V	The fundus is entirely hidden from view by the cloudy, grayish-white cortex and nucleus of the lens, the mature cataract stage.	7

### Lens structure and protein thermal stability are disrupted

The HE staining results presented in Figure 3B revealed significant disruptions to the lens structure and cellular organization in the model group, as compared to the normal group. Specifically, the normal group (3B-a) exhibited neatly arranged, single-layered lens epithelial cells with clear nuclear structure, as well as well-organized and regular fiber cells. In contrast, the model group (3B-b) showed no clear monolayer epithelial cell structure, with multi-layered nuclear structure and nuclei appeared in the fiber cell area. Moreover, there was no discernible fiber cell structure in this group, and the fiber cell structure was disrupted with vacuoles. In areas without vacuoles, the model group (3B-c) had a single-layered lens epithelial cell structure, although part of the nuclear structure was unclear, and the structure of the fiber cells was irregular and disordered. Additionally, nuclei were present in the fiber cell area in this group as well.

The findings revealed that whereas the OD value of the normal group continuously increased from 37° C to 86° C, the cataract group’s supernatant became increasingly cloudy at a faster rate than the normal group from 62° C to 86° C, and the trend became more pronounced as the temperature rose (Figure 3C). The supernatant of the protein at 86° C is shown in Figure 3D.

### CRYAA expression was decreased in rabbit lens

According to the PCR results (Figure 3E) and Western Blotting results (Figure 3F, 3G), the lens of cataract group had lower expression levels of the CRYAA mRNA (*p* value: 0.0031) and protein expression (*p* value: 0.0117) compared to the normal group.

### Selection of plasmids

**Figure 4:**
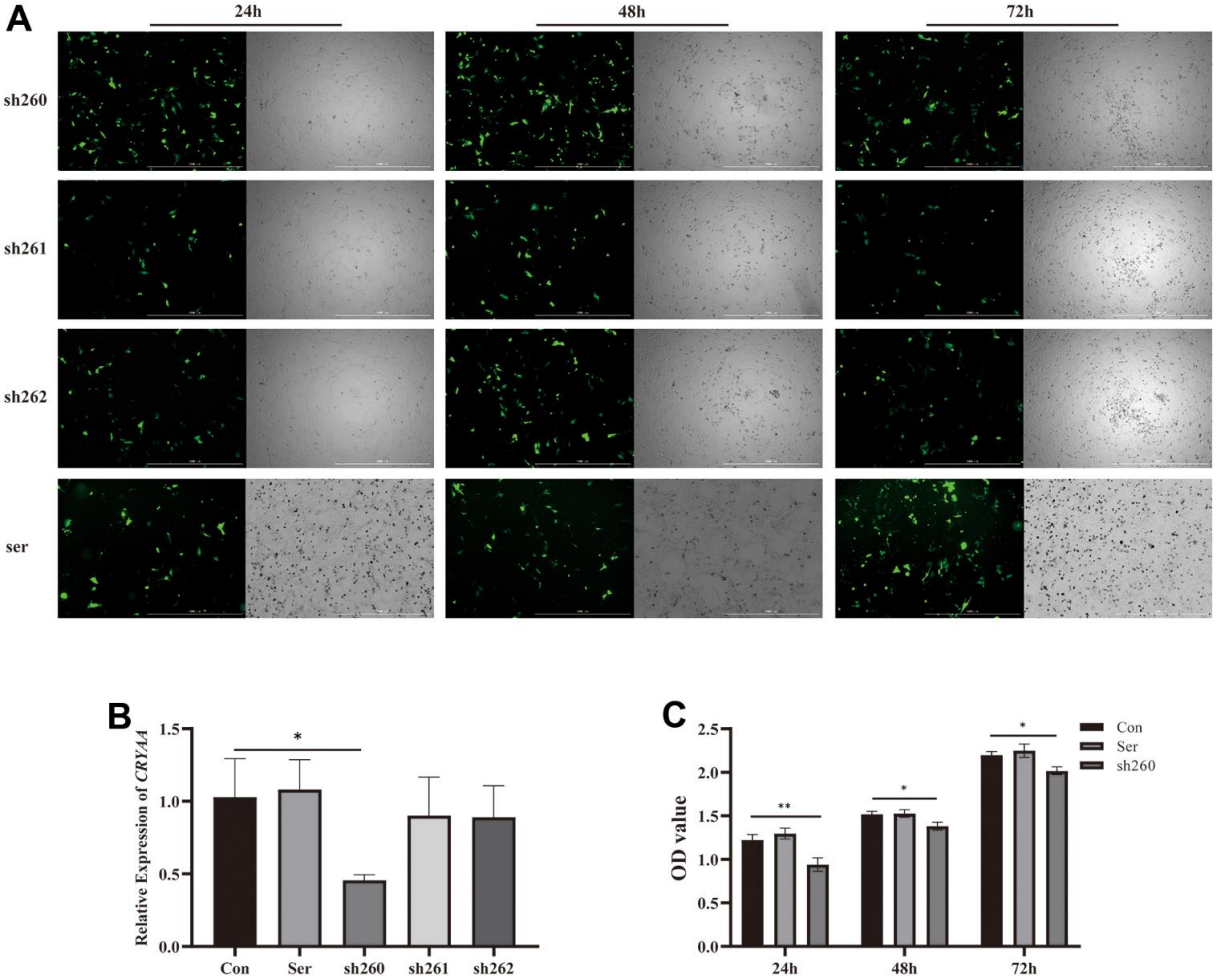
**Plasmid transfection efficiency and detection of its activity.** (**A**) Fluorescence of plasmid transfection at 24,48,72 h; (**B)** 48 hours sh260 CRYAA expression amount compared with the normal group, with statistical significance, * *p*<0.05. (**C**) Cell activity at three time periods 24, 48, and 72 after transfection, OD value of sh260 group compared with the normal group, with statistical significance, ** *p*<0.05, * *p*<0.05. There was no difference between ser and con group.

Based on the results of cell and animal experiments, we have found that the expression of CRYAA is decreased in the ARC models. This suggests that the decrease of CRYAA may be related to ARC. Therefore, we decided to silence CRYAA in HLEB3 cells to investigate its effect on cell physiological functions. To do this, we transfected three plasmids in to cells for 24, 48, and 72 hours using Lipo3000 transfection reagent and took pictures. The results are shown in Figure 4A. We found that the fluorescence of the transfected cells was strongest at 48 h, and the cells appeared to be in good condition.

PCR was then performed 48 hours after cells transfection to measure CRYAA content, and sh260 showed the best silencing efficiency among the three experiments (Figure 4B, *p* value: 0.0277), thus we selected it for further experiments. Additionally, CCK8 assays were conducted at 24, 48 and 72 hours after cells transfection, which showed a relative reduction in cell activity in the sh260 group (Figure 4C, p value: 0.0041, 0.0127, 0.0126) compared to the control group.

### Silencing of CRYAA promoted cell apoptosis and autophagy

**Figure 5:**
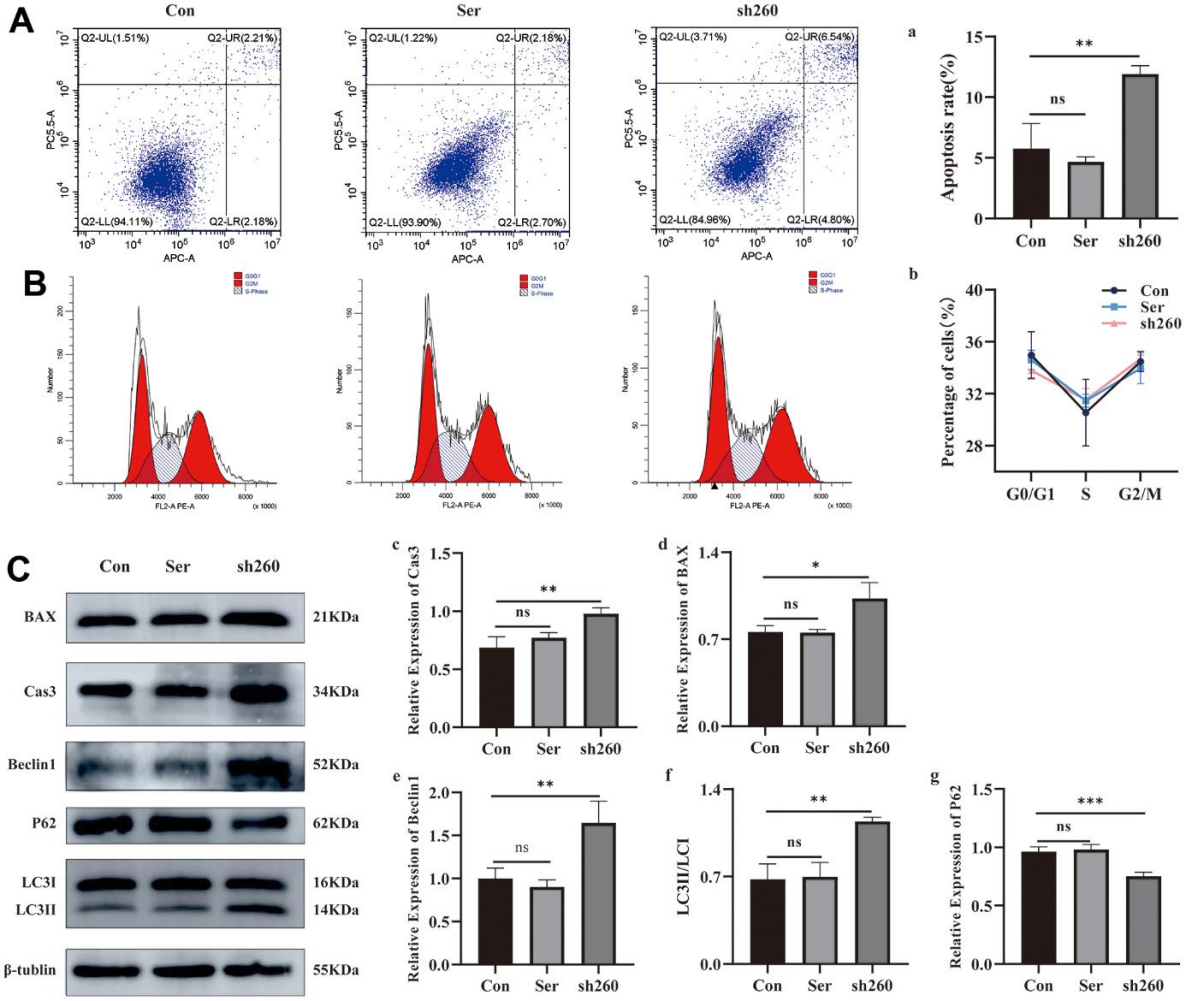
**Effect of CRYAA silencing on apoptosis and autophagy.** (**A**, a) Flow cytometry results of apoptosis at 48 h after transfection, sh260 group compared with the normal group, with statistical significance, ** *p*<0.01; (**B**, b) CRYAA silencing had no effect on the cell cycle. (**C**) WB results for apoptosis and autophagy proteins, c and d are the result of the apoptotic proteins Cas3 and Bax, sh260 group compared with the normal group, with statistical significance, ** *p*<0.01, * *p*<0.05. e and g are the result of the autophagy proteins Beclin1and P62, f is the result of LC3II/LC3I, sh260 group compared with the normal group, with statistical significance, *** *p*<0.01, ** *p*<0.01. There was no difference between ser and con group.

Based on the flow cytometry data, we found that silencing CRYAA had no effect cell cycle of HLEB3 cells (Figure 5B, 5b). However, it did promote cell apoptosis (Figure 5A, 5a) compared with the normal group, with statistical significance (*p* value: 0.0019) in the sh260 group. Additionally, depletion of CRYAA was further confirmed to promote cell apoptosis through the detection the apoptotic proteins CASP 3 and BAX (Figure 5C, 5c, 5d (*p* value: 0.0031, 0.0114)). Furthermore, the increase of Beclin1, the increase of LC3II/LC3I ratio and the decrease of P62 indicated that depletion of CRYAA promoted autophagy (Figure 5C, 5e, 5f, 5g (*p* value: 0.0059, 0.0022, 0.0009)).

## DISCUSSION

Lens opacity is the primary symptom of ARC, which is the world’s most common cause of blindness. Protein precipitation and aggregation, which occur due to the low stability of lens proteins, are the main causes of lens opacity [9, 17]. αA-crystallin plays a crucial role in maintain the stability of αB and β/γ-crystallin to ensure the transparency of the lens. It functions as a chaperone in the lens, binding partially denatured proteins to keep them soluble *in vitro* and maintain lens transparency [17]. Mice lacking the αA-crystallin gene will eventually develop cataracts, and inclusion bodies containing αB- and γ-crystallin aggregates form in the lens [18]. When stimulates with NaIO_3_, intracellular reactive oxygen species in these cells can be markedly elevated, while the cellular antioxidant capacity can be markedly reduced [19].

In this *in vitro* investigation, H_2_O_2_ was shown to enhance the apoptosis of HLEB3 cells, which is similar to the mechanism by which oxidative stress induces apoptosis in lens epithelial cells in ARC. Moreover, H_2_O_2_ was found to significantly impact cell growth and disrupt the regular cell cycle of HLEB3. PCR and Westering Blotting results revealed that CRYAA expression was decreased in HLEB3 cells following H_2_O_2_ intervention, suggesting that decreased CRYAA expression in HLEB3 cells may induce cataract development. Previous studies found CRYAA would be over-expressed in lens epithelial cells to help cells resist the damage induced by H_2_O_2_ and play an anti-apoptosis role [20, 21]. Additionally, the expression of the CRYAA gene in Human lens epithelial cells and lens epithelial cell lines was found to increase after UV exposure, but it returned to normal levels after the cell passage [22], suggesting that the increase in CRYAA is a secondary protective mechanism brought on by oxidative stress in lens epithelial cells. Therefore, when oxidative stress was removed, CRYAA expression returns to normal levels.

In studies involving animals, naphthalene was used to create a rabbit ARC model and produced disruption to the structure of the rabbit lens, as evidenced by both slit-lamp images and HE data. The results of PCR and Western Blotting revealed that CRYAA expression both dropped in rabbits with cataracts, suggesting that naphthalene cataract may harm the CRYAA gene and diminish its protein expression. Related studies have shown that the proportion of α-crystallin that is not bound to denatured proteins in clear lenses decreases by about 6-fold with age, and this proportion continues to increase with increasing nuclear opacity grade in cataract patients, with almost no free α-crystallin is present in nuclear cataract lenses with grade greater than 2 [23]. As people age, the ratio of αA- to αB-crystallin in α-crystallin decreases from 3:1 to 3: 2 [24]. Age-related nuclear cataract patients have lower CRYAA levels in their nuclei compared to the lens of the age-related healthy control group, and this decrease becomes more pronounced as the cataract progresses from grade II to grade IV [25, 26]. The amount of CRYAA in the lens can be decreased by hypermethylation of the CRYAA promoter region [26]. Some studies found the transcriptional activity of the CRYAA promoter can be decreased by the SNP located at rs7278468 [27]. Additionally, the CRYAA protein exon 1 mutation G>A at location 6 is more likely to result in nuclear age-related cataract [28].

In order to identify the CLA function of CRYAA, the protein thermostability was chosen for this experiment. Because at high temperatures, the recombinant αA-crystallin displayed a greater CLA than the recombinant αB-crystallin [29, 30]. The findings indicated that the thermal stability of protein declined in the cataract rabbit lens, which may obliquely demonstrate that the function of αA-crystallin CLA is diminished in cataract lenses. This experimental result may be related to the decrease or saturation of CRYAA chaperone activity, which may be the potential mechanism of cataract occurrence and age-related disorders in individuals over the age of 50 [26, 31]. In the lenses of naphthalene cataract rats with reduced α-crystallin CLA, post-translational changes such as C-terminal truncation of the 16 amino acids of α-crystallin, as well as acetylation, phosphorylation, oxidation, and carbonylation, were found [5]. One of the Single Nucleotide Polymorphisms (SNPS) linked to ARC is the 71th C>A mutation in exon 2 of the CRYAA gene, which causes an almost total loss of CLA (90%) [26].

Another mutation, the rs76740365 G>A mutation in exon 3 harbors of the CRYAA gene, alters the protein’s surface hydrophobicity and enhances its CLA activity. This mutation may also make the protein anti-apoptotic by down-regulating CASP3 and activating the p-AKT signaling pathway [32].

αA-crystallin is not only a molecular chaperone but also an antiapoptotic protein. A study shows that CRYAA inhibits apoptosis by enhancing PI3K activity and inactivating phosphatase tensin homologue, and the antiapoptotic function is directly related to its chaperone activity [33]. Our experiments showed that knockdown of CRYAA could reduce cell viability and induce apoptosis in HLEB3 cells. Recent study has shown that αA-crystallin is involved in the progress of proteasomal degradation of misfolded proteins in the endoplasmic reticulum (ER) [34]. CRYAA can bind unfolded, aggregation-prone substrates to retain them in solution [34]. Endoplasmic reticulum stress (ERS) and unfolded protein response (UPR) are induced when the body is exposed to adverse external stimuli like ROS, which has been established that can induce different cell death modes, including autophagy, apoptosis [35]. A study showed that prolonged activation of the unfolded protein reaction (UPR) pathway and severe stress response can cause proteotoxic and endoplasmic reticulum stress (ERS), which induced cell death in CRYAA-Y118D mutant mice [36]. Autophagy is a fundamental cellular requirement for achieving the mature structure, homeostasis, and transparency of the lens [14]. However, in the aging process of human LECs, oxidative stress sets off the long-lasting activation of autophagy and causes excessive P62 protein degradation, both of which contribute to early senescence. Low levels of P62, a scaffold protein, cannot maintain adequate pro-survival signaling PKCι-IKK-NF-κB cascades, aggravating oxidative stress-induced apoptosis [37]. We found that depletion of CRYAA could promote the occurrence of autophagy accompanied by the low expression of P62. These indicates that apoptosis and excessive autophagy caused by CRYAA decreased may be one of the causes of ARC formation.

In conclusion, this study examined the effects of two common ARC modeling methods on CRYAA *in vivo* and *in vitro*, and the results were consistent with clinical results of ARC. Furthermore, it was found that HLEB3 cells promoted cell apoptosis and excessive autophagy, which led to premature senescence after CRYAA silencing. This cell model could potentially serve as a model for ARC, in addition to external ROS stimulation. We are committed to elucidating the expression of CRYAA in ARC model and its role in LECs, hoping to help better understand the etiology of ARC and make a contribution to the research of ARC.

## MATERIALS AND METHODS

### Experimental methods and animals

New Zealand white rabbits (six-month-old, half male and half female), purchased from the Animal Experimental Center of Ningxia Medical University. The rabbits were kept in a normal laboratory environment with a 12-hour light and dark cycle. The diet was provided by the laboratory. Before experiment, their eyes were examined with a slit lamp (Suzhou Kangjie, CHN) to check for ophthalmic diseases and lens opacity. Rabbits with normal lenses were screened. Ten rabbits were randomly assigned to each group, with half being male and half being female.

### Establishment of cataract rabbit model

On the basis of the artificial model method [2, 6], 30g naphthalene (Cas:91-20-3, Shanghai Guangnuo, CHN) was dissolved in 100ml of Tween-80 (Cas:9005-65-6, Tianjin Hengxing, CHN) solution, and a 30% suspension was prepared. The suspension was sonicated in a water-bath ultrasonic pan for 1 hour to ensure the naphthalene to dissolved completely in Tween-80. After allowing the naphthalene to settle and precipitate, the ultrasound was repeated 2-3 times. After standing, naphthalene precipitates into a fine powder (ensure that it does not block the feeding tube).

The rabbits in the model group were administered naphthalene at 0.8 g/kg/day by intragastric administration. Initially, they were given the treatment every 3 days and allowed 1 day of rest. This continued for 1 week. On the other hand, the rabbits in the control group were treated with the same volume of Tween-80. The rabbits were treated with mydriasis (compound tropicamide), and the lens was observed by a slit lamp every 3-4 days, dilate the pupil with Tropicamide Phenylephrine Eye Drops (J20180051, Santen Pharmaceutical, CHN). When cataracts occurred in the model group, naphthalene administration was discontinued, and rabbits without cataracts were excluded from further analysis. When all remains rabbits in the model group reached IV-V cataract, the rabbits were sacrificed after air embolism in the ear vein, and the lens was removed.

### Hematoxylin and eosin staining (HE)

The lens tissues were fixed in a 4% paraformaldehyde solution (D16013, Shangbao, CHN), then removed, dehydrated using a gradient concentration of alcohol, and immersed in xylene. They were then embedded in wax, then sectioned, glued, deparaffinized, alcoholized, and stained with hematoxylin and eosin. Finally, the lens tissues were observed and photographed under a microscope.

### Cell cultural and plasmid transfection

HLEB3 cells (BNCC 30494, Beijing Beina Chuanglian Institute of Biotechnology.) were removed from the liquid nitrogen tank, resuscitated with medium (containing 89% MEM medium, 10% FBS and 1% double antibody), and cultured to an incubator (culture condition: 37° C, 5% CO_2_). The medium was changed every 2-3 days, and the cells were passaged when their density reached 80-90%.

shRNA fragments of CRYAA included shRNA-260, shRNA-261, shRNA-262 and negative control (ser), were obtained from RiboBio, CHN, which can Silence the expression of *CRYAA*. HLEB3 cells were inoculated into 6-well plates. 24 h later, according to the transfection instructions, the cell medium was replaced with the medium containing Lipofectamine 3000 (L3000008, Invitrogen, USA) transfection reagent, corresponding shRNA and MEM, and 6 h after transfection, the medium was replaced with the complete medium. Fluorescence was taken at 24, 48 and 72 hours by cytation5 (Biotek, USA).

### The cell counting Kit-8 (CCK8) method detected cell viability

The cells were divided into three groups: normal group, negative control group and plasmid transfection group. Each group contained 10,000 cells per well, and the cells were seeded into 96-well plates. Cell activity was detected using the CCK8 assay for 24, 48 and 72 hours, respectively.

### Cell apoptosis and cell cycle were detected by flow cytometry

The HLEB3 cells were seeded at a density of 2×10^5^ cells/well in 6-well plates. After allowing the cells to adhere and grow to 70-80% confluency, two groups were established: a “normal group” with no treatment, and a “model group” treated with 500μmol/L hydrogen peroxide (30%, Cas:7722-84-1, Yantai Shuangshuang, CHN) for X hours. Then cells assayed at 12, 24, and 36 hours by BD AccuriTM C6 Software (Becton, Dickinson and Company, USA). Annexin V-FITC/PI apoptosis detection kit (KGA107, KeyGEN, CHN); Cell cycle detection kit (KGA512, KeyGEN, CHN).

The selected plasmids were transfected into cells for apoptosis (Annexin V-APC/7-AAD Apoptosis Detection Kit, KGA1025, KeyGEN, CHN) and cell cycle analysis by CytoFLEX S (Beckman Coulter, Inc. USA).

### Real-time fluorescence quantitative polymerase chain reaction (PCR)

The following cells or tissues were selected for *CRYAA* mRNA content detection: A. The HLEB3 cells were treated with 0, 500μmol/L hydrogen peroxide for 12, 24, 36 hours; B. The HLEB3 cells were treated with 0, 300, 500, 700μmol/L hydrogen peroxide for 24 hours; C. Normal rabbit lens and cataract rabbit lens; D. The transfected cells 48 hours which with good fluorescence intensity was selected to screen out the shRNA with the best transfection efficiency. Using RNA extraction reagent trizol (ThermoFisher, USA) extracted total RNA from cells and tissues, TB Green^®^ Premix Ex Taq^™^ II (Tli RNaseH Plus) (RR820A, Takara, JPN) and PrimeScript^™^ RT reagent Kit with gDNA Eraser (Perfect Real Time) (RR047A, Takara, JPN) were then used for follow-up experiments. PCR was performed on Fluorescence quantitative PCR instrument (StepOne Software V2.3, ThermoFisher, USA) for 40 cycles, and the *CRYAA* mRNA expression was analyzed by the 2^-ΔΔCт^ method. The sequence of primers: human *CRYAA* (Forward 5’- TCCAGCACCCCTGGTTCAAG -3’, Reverse 5’- CTTGTCCCGGTCGGATCGAA -3’), human GAPDH (Forward 5’- ACACCATGGGGAAGGTGAAG -3’, Reverse 5’ –TAGGGGTCATTGATGGCAAC -3’); rabbit CRYAA (Forward 5’-GGACAAGTTCGTCATCTTCCTGGAC-3’, Reverse 5’-TGTAGCCGTGGTCATCCTGTCTC-3’), rabbit β-actin (Forward 5’-GTGGCATCCTGACGCTCAAGTAC-3’, Reverse 5’- AAGCTCGTTGTAGAAGGTGTGGTG-3’).

### Western blotting (WB)

The following cells or tissues were selected for CRYAA protein content detection: A. HLEB3 cells were treated with 0, 500μmol/L hydrogen peroxide for 12, 24, 36 hours; B. HLEB3 cells were treated with 0, 300, 500, 700μmol/L hydrogen peroxide for 24 hours; C: Normal rabbit lens and cataract rabbit lens; D. The cells that were transfected with plasmids were selected. The expression levels of BAX, CASPASE3, P62, Beclin1, LC3 were measured in plasmid transfected cells. Total protein was extracted from cells or tissues by the Whole protein extraction kit (KGP2100, KeyGEN, CHN), and the protein concentration was determined using the BCA kit (ZJ102, Epizyme, CHN). The samples were loaded at 40μg protein per lane for polyacrylamide gel electrophoresis and the membrane transfer. After the membranes were closed using 5% skim milk at room temperature for 2 hours, the primary antibodies were added and incubated at 4° C overnight. After washing the membranes, the secondary antibodies were added and incubated at room temperature for 2 hours. Electrophoresis apparatus (Bio-Rad, USA); Ultra-sensitive multi-function Imager (Amersham Imager 680RGB, USA); Protein ladder (No.PR1910, ThermoFisher, USA); measured gray value: Image J 1.48V(Wayne Rasband, National Institutes of Health, USA).

Antibodies: CRYAA antibody (DF8933, Affinity, USA); GAPDH (ab8245, Abcam, USA); β-actin (AF7018, Affinity, USA); Goat anti-rabbit (S0001, Affinity, USA); Goat anti-Mouse (S0002, Affinity, US); BAX (60267-1-Ig, Wuhan Sanying, CHN), CASPASE3 (ab13847, Abcam, US); P62 (ab56416, Abcam, US); BECN1(ab62557, Abcam, USA); LC3 (ab221794, Abcam, USA); β-tublin (T0023, Affinity, USA).

### Protein thermostability assay

An appropriate amount of normal and cataract rabbit lens tissue was added to 1ml of protein lysate (10μL PMSF per 1mL RIPA was added to make the final concentration of PMSF 1mM, PMSF was added on the spot). The sample was homogenized using a frozen grinder and then centrifuged at 12000g for 5 minutes at 4° C. The supernatant was then diluted, and protein concentration was determined at 560nm using BCA method, and the protein supernatant of each group was adjusted to the same concentration (5μg/μL). The same concentration of crystallin supernatant (100μL/ well) was added into the 96-well plate, and the constant temperature incubator was preheated to 37° C. The absorbance value was measured at 320 nm wavelength 37° C, with double distilled water used as reference. The absorbance value at the wavelength of 320nm was measured once every 6° C rise from 50° C for 3 minutes until the temperature reached 86° C.

### Statistical analyses

The data were expressed as numbers or percentage or mean ± standard error. One-way analysis of variance was used in dates of three or four groups, and t-test was used in dates of two groups. All data were analyzed using GraphPad Prism 8.2.0 (GraphPad software Inc., San Diego, California, USA). *p* < 0.05 was of statistical significance.

### Data availability

All of the data is available from the corresponding author upon reasonable request.

## References

[1] Cicinelli MV, Buchan JC, Nicholson M, Varadaraj V, Khanna RC. Cataracts. Lancet. 2023; 401:377–89. 10.1016/S0140-6736(22)01839-6 36565712

[2] Feng K, Guo HK. Eaf2 protects human lens epithelial cells against oxidative stress-induced apoptosis by Wnt signaling. Mol Med Rep. 2018; 17:2795–802. 10.3892/mmr.2017.8246 29257273 PMC5783493

[3] Chang L, Bao Y, Li X, Xiao L. Effects of JNK-signaling pathway in hyperbaric oxygen and H_2_O_2_-induced cellular apoptosis of human lens epithelial cells. Recent Advances in Ophthalmology. 2014; 34:923–6.

[4] Chen Y, Jiang Y, Yang J, Lu Y. Effect of calpain II on oxidative damage in naphthalene-induced cataract. Chinese Journal of Ophthalmology and Otorhinolaryngology. 2014; 14:273–7.

[5] Chen Y, Yi L, Yan GQ, Jang YX, Fang YW, Wu XH, Zhou XW, Wei LM. Decreased chaperone activity of alpha-crystallins in naphthalene-induced cataract possibly results from C-terminal truncation. J Int Med Res. 2010; 38:1016–28. 10.1177/147323001003800328 20819438

[6] Singh A, Bodakhe SH. Resveratrol delay the cataract formation against naphthalene-induced experimental cataract in the albino rats. J Biochem Mol Toxicol. 2020; 34:e22420. 10.1002/jbt.22420 31746523

[7] Pandya U, Saini MK, Jin GF, Awasthi S, Godley BF, Awasthi YC. Dietary curcumin prevents ocular toxicity of naphthalene in rats. Toxicol Lett. 2000; 115:195–204. 10.1016/s0378-4274(00)00191-0 10814889

[8] Rathbun WB, Holleschau AM, Cohen JF, Nagasawa HT. Prevention of acetaminophen- and naphthalene-induced cataract and glutathione loss by CySSME. Invest Ophthalmol Vis Sci. 1996; 37:923–9. 8603877

[9] Lee CM, Afshari NA. The global state of cataract blindness. Curr Opin Ophthalmol. 2017; 28:98–103. 10.1097/ICU.0000000000000340 27820750

[10] Selivanova OM, Galzitskaya OV. Structural and Functional Peculiarities of α-Crystallin. Biology (Basel). 2020; 9:85. 10.3390/biology9040085 32340218 PMC7235859

[11] Srinivas P, Narahari A, Petrash JM, Swamy MJ, Reddy GB. Importance of eye lens α-crystallin heteropolymer with 3:1 αA to αB ratio: stability, aggregation, and modifications. IUBMB Life. 2010; 62:693–702. 10.1002/iub.373 20836128 PMC3615983

[12] Srinivasan AN, Nagineni CN, Bhat SP. alpha A-crystallin is expressed in non-ocular tissues. J Biol Chem. 1992; 267:23337–41. 10.1016/S0021-9258(18)50096-X 1429679

[13] Yang J, Zhou S, Guo M, Li Y, Gu J. Different alpha crystallin expression in human age-related and congenital cataract lens epithelium. BMC Ophthalmol. 2016; 16:67. 10.1186/s12886-016-0241-1 27234311 PMC4884376

[14] Brennan L, Costello MJ, Hejtmancik JF, Menko AS, Riazuddin SA, Shiels A, Kantorow M. Autophagy Requirements for Eye Lens Differentiation and Transparency. Cells. 2023; 12:475. 10.3390/cells12030475 36766820 PMC9914699

[15] Weinberg J, Gaur M, Swaroop A, Taylor A. Proteostasis in aging-associated ocular disease. Mol Aspects Med. 2022; 88:101157. 10.1016/j.mam.2022.101157 36459837 PMC9742340

[16] Zhang Z. Establishment Three Kinds Models of Cataract and Study on AQP0 Expression in Oxidative Cataract in Canine. Southwest University. 2015.

[17] Horwitz J. Alpha-crystallin. Exp Eye Res. 2003; 76:145–53. 10.1016/s0014-4835(02)00278-6 12565801

[18] Brady JP, Garland D, Duglas-Tabor Y, Robison WG Jr, Groome A, Wawrousek EF. Targeted disruption of the mouse alpha A-crystallin gene induces cataract and cytoplasmic inclusion bodies containing the small heat shock protein alpha B-crystallin. Proc Natl Acad Sci USA. 1997; 94:884–9. 10.1073/pnas.94.3.884 9023351 PMC19608

[19] Michael R, Bron AJ. The ageing lens and cataract: a model of normal and pathological ageing. Philos Trans R Soc Lond B Biol Sci. 2011; 366:1278–92. 10.1098/rstb.2010.0300 21402586 PMC3061107

[20] Zhou P, Ye HF, Jiang YX, Yang J, Zhu XJ, Sun XH, Luo Y, Dou GR, Wang YS, Lu Y. αA crystallin may protect against geographic atrophy-meta-analysis of cataract vs. cataract surgery for geographic atrophy and experimental studies. PLoS One. 2012; 7:e43173. 10.1371/journal.pone.0043173 22916220 PMC3423426

[21] Su S, Liu P, Zhang H, Li Z, Song Z, Zhang L, Chen S. Proteomic analysis of human age-related nuclear cataracts and normal lens nuclei. Invest Ophthalmol Vis Sci. 2011; 52:4182–91. 10.1167/iovs.10-7094 21436267

[22] Li D, Luo Y, Lu Y. Ultraviolet C induced up-regulation expression of CRYAA in human lens epithelial cells. Chinese Journal of Ophthalmology and Otorhinolaryngology. 2017; 17:239–44.

[23] Shiels A, Hejtmancik JF. Mutations and mechanisms in congenital and age-related cataracts. Exp Eye Res. 2017; 156:95–102. 10.1016/j.exer.2016.06.011 27334249 PMC5538314

[24] Ma Z, Hanson SR, Lampi KJ, David LL, Smith DL, Smith JB. Age-related changes in human lens crystallins identified by HPLC and mass spectrometry. Exp Eye Res. 1998; 67:21–30. 10.1006/exer.1998.0482 9702175

[25] Zhou P, Luo Y, Liu X, Fan L, Lu Y. Down-regulation and CpG island hypermethylation of CRYAA in age-related nuclear cataract. FASEB J. 2012; 26:4897–902. 10.1096/fj.12-213702 22889833

[26] Bhagyalaxmi SG, Srinivas P, Barton KA, Kumar KR, Vidyavathi M, Petrash JM, Bhanuprakash Reddy G, Padma T. A novel mutation (F71L) in alphaA-crystallin with defective chaperone-like function associated with age-related cataract. Biochim Biophys Acta. 2009; 1792:974–81. 10.1016/j.bbadis.2009.06.011 19595763 PMC3816373

[27] Ma X, Jiao X, Ma Z, Hejtmancik JF. Polymorphism rs7278468 is associated with Age-related cataract through decreasing transcriptional activity of the CRYAA promoter. Sci Rep. 2016; 6:23206. 10.1038/srep23206 26984531 PMC4794711

[28] Bhagyalaxmi SG, Padma T, Reddy GB, Reddy KR. Association of G>A transition in exon-1 of alpha crystallin gene in age-related cataracts. Oman J Ophthalmol. 2010; 3:7–12. 10.4103/0974-620X.60014 20606865 PMC2886234

[29] Kumar MS, Kapoor M, Sinha S, Reddy GB. Insights into hydrophobicity and the chaperone-like function of alphaA- and alphaB-crystallins: an isothermal titration calorimetric study. J Biol Chem. 2005; 280:21726–30. 10.1074/jbc.M500405200 15817465

[30] Reddy GB, Das KP, Petrash JM, Surewicz WK. Temperature-dependent chaperone activity and structural properties of human alphaA- and alphaB-crystallins. J Biol Chem. 2000; 275:4565–70. 10.1074/jbc.275.7.4565 10671481

[31] Shroff NP, Cherian-Shaw M, Bera S, Abraham EC. Mutation of R116C results in highly oligomerized alpha A-crystallin with modified structure and defective chaperone-like function. Biochemistry. 2000; 39:1420–6. 10.1021/bi991656b 10684623

[32] Zhao Z, Sun Y, Fan Q, Jiang Y, Lu Y. Structural and functional analysis of SNP rs76740365 G>A in exon-3 of the alpha A-crystallin gene in lens epithelial cells. Mol Vis. 2022; 28:317–30. 10.21203/rs.3.rs-1399398/v1 36338667 PMC9603911

[33] Pasupuleti N, Matsuyama S, Voss O, Doseff AI, Song K, Danielpour D, Nagaraj RH. The anti-apoptotic function of human αA-crystallin is directly related to its chaperone activity. Cell Death Dis. 2010; 1:e31. 10.1038/cddis.2010.3 21364639 PMC3032290

[34] Kashlan OB, Mueller GM, Qamar MZ, Poland PA, Ahner A, Rubenstein RC, Hughey RP, Brodsky JL, Kleyman TR. Small heat shock protein alphaA-crystallin regulates epithelial sodium channel expression. J Biol Chem. 2007; 282:28149–56. 10.1074/jbc.M703409200 17664274 PMC2361386

[35] Zhang J, Guo J, Yang N, Huang Y, Hu T, Rao C. Endoplasmic reticulum stress-mediated cell death in liver injury. Cell Death Dis. 2022; 13:1051. 10.1038/s41419-022-05444-x 36535923 PMC9763476

[36] Jia ZK, Fu CX, Wang AL, Yao K, Chen XJ. Cataract-causing allele in CRYAA (Y118D) proceeds through endoplasmic reticulum stress in mouse model. Zool Res. 2021; 42:300–9. 10.24272/j.issn.2095-8137.2020.354 33929105 PMC8175955

[37] Huang J, Yu W, He Q, He X, Yang M, Chen W, Han W. Autophagy facilitates age-related cell apoptosis-a new insight from senile cataract. Cell Death Dis. 2022; 13:37. 10.1038/s41419-021-04489-8 35013122 PMC8748728

